# Assessment of RACGAP1 as a Prognostic and Immunological Biomarker in Multiple Human Tumors: A Multiomics Analysis

**DOI:** 10.3390/ijms232214102

**Published:** 2022-11-15

**Authors:** Refaat A. Eid, Mohamed A. Soltan, Muhammad Alaa Eldeen, Ayed A. Shati, Samy A. Dawood, Mohamed Eissa, Mohamed Samir A. Zaki, Mohammad Algahtani, Abdulrahman Theyab, Mohamed M. Abdel-Daim, Bonglee Kim

**Affiliations:** 1 Pathology Department, College of Medicine, King Khalid University, Abha P.O. Box 62529, Saudi Arabia; 2 Department of Microbiology and Immunology, Faculty of Pharmacy, Sinai University, Ismailia 41611, Egypt; 3 Cell Biology, Histology & Genetics Division, Biology Department, Faculty of Science, Zagazig University, Zagazig 44519, Egypt; 4 Department of Child Health, College of Medicine, King Khalid University, Abha P.O. Box 62529, Saudi Arabia; 5 Clinical Pathology Department, Faculty of Medicine, Zagazig University, Zagazig 44519, Egypt; 6 Anatomy Department, College of Medicine, King Khalid University, Abha P.O. Box 62529, Saudi Arabia; 7 Department of Histology and Cell Biology, College of Medicine, Zagazig University, Zagazig 31527, Egypt; 8 Department of Laboratory & Blood Bank, Security Forces Hospital, Mecca P.O. Box 14799, Saudi Arabia; 9 College of Medicine, Al-Faisal University, Riyadh P.O. Box 50927, Saudi Arabia; 10 Department of Pharmaceutical Sciences, Pharmacy Program, Batterjee Medical College, Jeddah P.O. Box 6231, Saudi Arabia; 11 Pharmacology Department, Faculty of Veterinary Medicine, Suez Canal University, Ismailia 41522, Egypt; 12 Department of Pathology, College of Korean Medicine, Kyung Hee University, Seoul 02447, Republic of Korea

**Keywords:** RACGAP1, pan-cancer, phosphorylation, prognosis, tumor immunotherapy, biomarker

## Abstract

Several recent studies have pointed out that arc GTPase activating protein 1 (RACGAP1) is a putative oncogene in many human tumors. However, to date, no pan-cancer analysis has been performed to study the different aspects of this gene expression and behavior in tumor tissues. Here, we applied several bioinformatics tools to perform a comprehensive analysis for RACGAP1. First, we assessed the expression of RACGAP1 in several types of human tumors and tried to correlate that with the stage of the tumors analyzed. We then performed a survival analysis to study the correlation between RACGAP1 upregulation in tumors and the clinical outcome. Additionally, we investigated the mutation forms, the correlation with several immune cell infiltration, the phosphorylation status of the interested protein in normal and tumor tissues, and the potential molecular mechanisms of RACGAP1 in cancerous tissue. The results demonstrated that RACGAP1, a highly expressed gene across several types of tumors, correlated with a poor prognosis in several types of human cancers. Moreover, it was found that RACGAP1 affects the tumor immune microenvironment by influencing the infiltration level of several immune cells. Collectively, the current study provides a comprehensive overview of the oncogenic roles of RACGAP1, where our results nominate it as a potential prognostic biomarker and a target for antitumor therapy development.

## 1. Introduction

The process of tumorigenesis has proven to be complex, involving a series of interactions between various genes that consequently transfer the cells from the normal state to the cancerous condition [1,2]. Hence, a deep study of different oncogenes is an essential process to explore the molecular mechanisms of several genes in cancer development [3]. A pan-cancer analysis that employs available databases such as The Cancer Genome Atlas (TCGA) gave us a great opportunity to analyze the expression and the behavior of a specific gene in a large list of tumors in an economical and time-saving approach with the currently developed bioinformatics tools [4,5].

RACGAP1, a constituent of the centralspindlin that is important for the activation of cytokinesis [6,7], seems to be a member of the Rho GTPase-activating protein family [8]. RACGAP1 coupled with GTP-bound Rac1 functions as both a mediating factor of tyrosine phosphorylation of the signal transducer and activator of transcription (STAT) family of proteins and as a nuclear localization signal-containing nuclear chaperone of phosphorylated STATs, which already have multiple functions, including antiapoptosis, proliferation, differentiation, and inflammation [9]. RACGAP1 also controls the activity of Rho proteins, including Rac and CDC42, to affect cell shape, migration, and polarization [10]. Concerning its subcellular distribution, RACGAP1 colocalizes with RND2 in proacrosomal vesicles produced from the Golgi and the acrosome. Throughout interphase, it is directed to the nucleus and cytoplasm alongside microtubules; during anaphase, it is organized to the central spindle; during telophase or even cytokinesis, it is concentrated to the midbody. During cytokinesis, RACGAP1 is colocalized with RhoA at the myosin contractile ring and ECT2 at the mitotic spindles during anaphase/metaphase, at the cleavage furrow during telophase, and to the midbody. Lastly, it interacts with CDC42 to spindle microtubules from prometaphase to telophase [11].

RACGAP1 is overexpressed in the testes, thymus, placenta, gastrointestinal system, urinary tract, uterus, cervix, skin, tonsil, nasopharynx, and bone marrow, as well as the lymphatic system [12]. It is present in lower concentrations in the spleen and peripheral blood lymphocytes. RACGAP1 expression is limited to germ cells in the testis, with the greatest level reported in spermatocytes [13,14]. Its expression is regulated throughout the cell cycle, peaking during the G2/M phase [15]. The RACGAP1 gene is conserved in chimpanzees, Rhesus monkeys, dogs, cows, mice, rats, chickens, zebrafish, fruit flies, mosquitoes, and frogs, as indicated by NCBI data [16].

Although it has been established that RACGAP1 plays an important role in the progression of different types of tumors, there are a lack of studies that analyze the collective action of RACGAP1 in a group of tumors, and for this purpose, we present here the first systematic pan-cancer analysis of RACGAP1. The current study analyzed the expression profile of RACGAP1 across several types of tumors and tried to correlate that with the prognosis and the infiltration of the immune cells. We also investigated *RACGAP1* gene mutation types besides the estimation of the interacted and correlated gene network. This comprehensive study demonstrates the predicted molecular roles of RACGAP1 in several cancer types in addition to its influence on clinical prognosis.

## 2. Results

The abbreviations and the full names of analyzed tumors in the current study are shown in Supplementary Table S1.

### 2.1. RACGAP1 Increased Expression in Many Tumor Types

**Figure 1:**
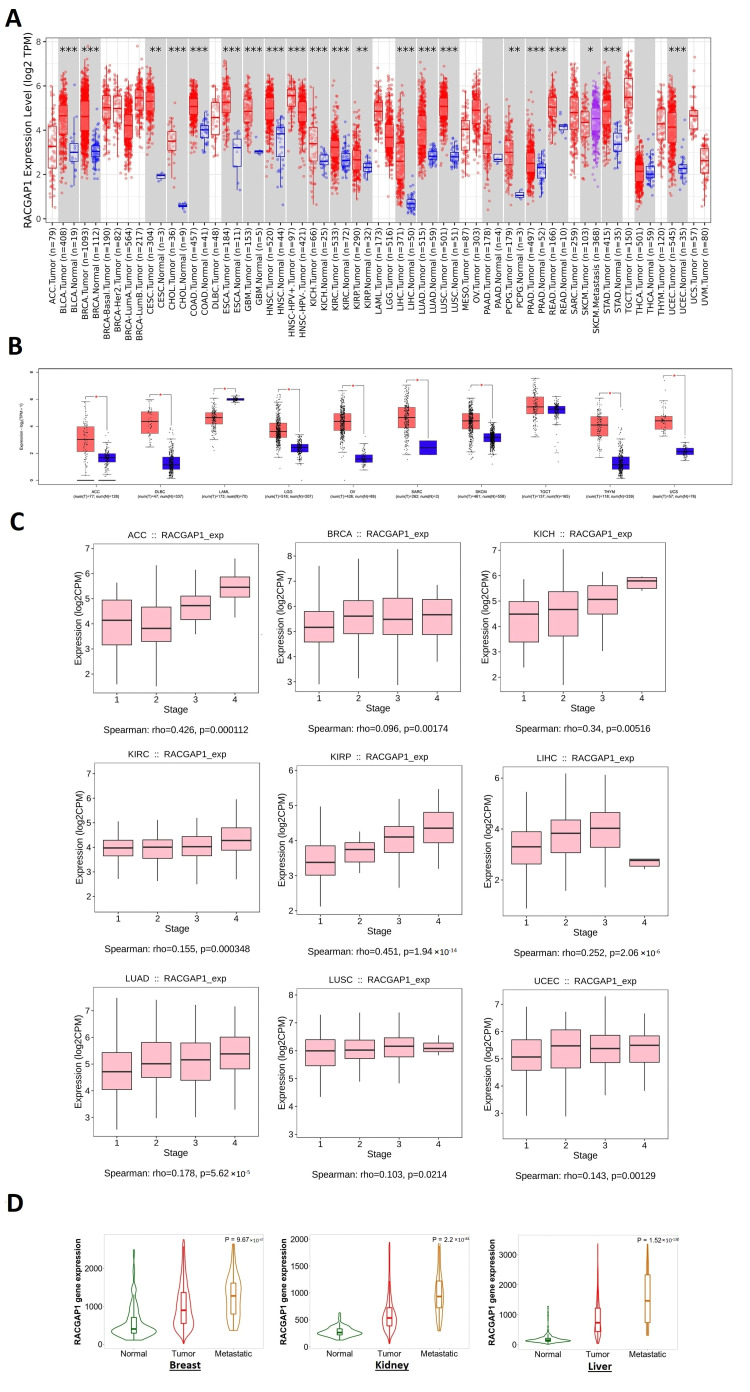
RACGAP1 expression assessment in human cancers. (**A**) Differential expression of RACGAP1 in a panel of TCGA tumors analyzed by TIMER2.0; (**B**) the tumors that lack normal tissue for comparison in TIMER2.0 database and analyzed in the GEPIA database; (**C**) tumors experienced a positive correlation between RACGAP1 expression and tumor stage when analyzed with the TISDIB web server; (**D**) tumors experienced a consistent positive correlation between RACGAP1 expression and tissue type (normal–tumor–metastatic); * means statistically significant *p* > 0.05, ** means high statistically significant *p* > 0.001 and *** means *p* > 0.0001.

Initially, TIMER2 was used to examine the differential expression of our target gene between malignant and neighboring healthy tissues. RACGAP1 was observed to be significantly overexpressed in several cancers (Figure 1A), including BLCA, BRCA, CHOL, COAD, ESCA, GBM, HNSC, KICH, KIRC, LIHC, LUAD, LUSC, PRAD, READ, STAD, as well as UCEC (*p* < 0.001), CESC, KIRP, and PCPG (*p* < 0.01). Due to the inaccessibility of normal tissue expressions to be used as a reference for some malignancies in TIMER2, we accessed the Interactive Gene Expression Profiling Analysis (GEPIA) database to acquire differential expression in normal and malignant tissues for these cancers. Most of the malignancies examined, including ACC, DLBC, LGG, O.V., SARC, SKCM, THYM, and UCS, exhibited a statistically significant upregulation of RACGAP1 in cancerous versus normal tissues (Figure 1B). On the contrary, only two cancers, TGCT and LAML, did not exhibit significant differences and upregulation of RACGAP1 in healthy tissues compared to malignant tissue, respectively.

Following examining the differential expression of RACGAP1 in normal and malignant tissues, we intended to investigate the association between RACGAP1 expression and cancer staging. Using the TISDIB web server, we determined that RACGAP1 expression was significantly associated with the disease stage of ACC, KIRC, KIRP, LIHC, LUAD (*p* < 0.001), BRCA, KICH, UCEC (*p* < 0.01), and LUSC (*p* < 0.05) (Figure 1C). The “compare tumor, normal, and metastasis” module of the TNMplot online server was then utilized to correlate the RACGAP1 mRNA expression levels with the metastases. RACGAP1 expression was significantly elevated in the breast, kidney, and liver when normal and tumor tissues were compared, and this pattern was also maintained between metastatic and cancerous tissues (Figure 1D).

### 2.2. Differential Protein Expression

**Figure 2:**
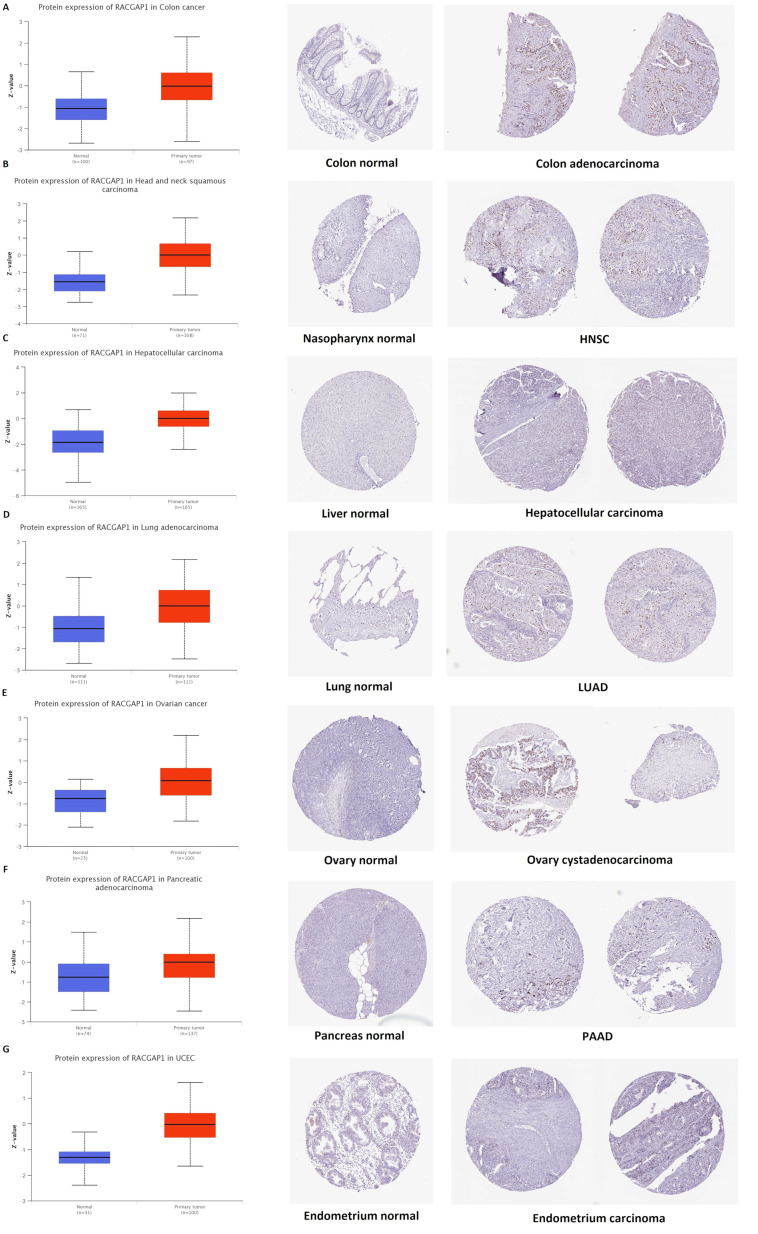
Tumors experienced a statistically significant higher RACGAP1 protein expression in the tumor sample versus normal one (**left** side), and IHC staining for normal tissue (**middle**) and cancerous one (**left**) demonstrated the same results. (**A**) Colon; (**B**) nasopharynx; (**C**) liver; (**D**) lung; (**E**) ovary; (**F**) pancreas; (**G**) endometrium.

Following investigating RACGAP1 at the transcriptional levels, we analyzed its protein level using the National Cancer Institute’s CPTAC dataset of large-scale proteome data. Figure 2A–G demonstrate that the expression of the RACGAP1 protein was significantly elevated in the colon, HNSC, hepatocellular carcinoma, LUAD, PAAD, UCEC, and colon cancer compared to normal tissues (*p* < 0.001). Subsequently, we retrieved the IHC figures for the normal and malignant tissues to validate our earlier results. Normal tissues of the colon, nasopharynx, liver, lung, ovary, pancreas, and endometrium did not exhibit any staining, so the findings were consistent. Similarly, it was moderate to high in the comparable malignant samples (Figure 2A–E).

### 2.3. Increased RACGAP1 Level Estimates Poor Clinical Outcomes

**Figure 3:**
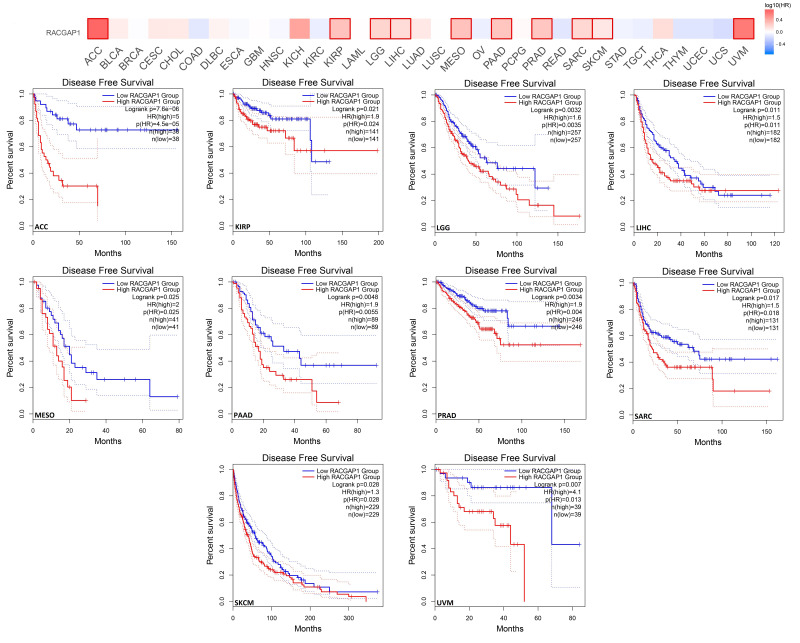
The correlation between RACGAP1 expression and the clinical outcome in a disease-free survival model as assessed from the GEPIA database.

To investigate the connection between RACGAP1 expression patterns and survival rates, we utilized GEPIA as well as Kaplan–Meier (KM) plotter databases. From GEPIA, we determined that the overexpression of our target gene is associated with poor disease-free survival (DFS) prognosis for ACC (*p* < 0.001), LGG, PAAD, PRAD, UVM (*p* < 0.01), KIRP, LIHC, MESO, and SARC, as well as SKCM (*p* < 0.05) (Figure 3). Furthermore, analysis of patients’ overall survival (OS) revealed that in addition to ACC, KIRP, LGG, LIHC, MESO, PAAD, SARC, and SKCM patients with a poor prognosis (similar to the disease-free survival model), LUAD (*p* < 0.001) patients also had a poor survival with the overexpression of RACGAP1 (Figure S1).

**Figure 4:**
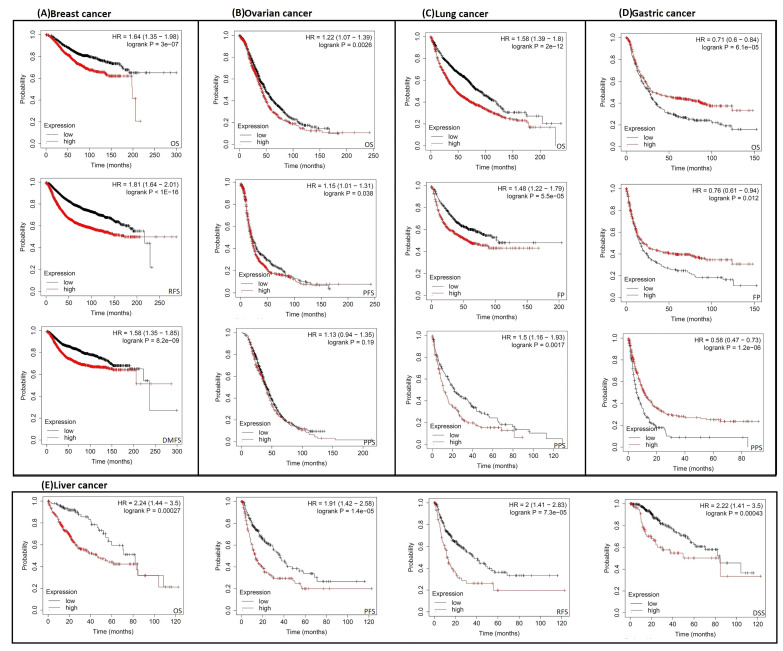
The correlation between RACGAP1 expression and the survival prognosis as assessed with the Kaplan–Meier plotter tool for (**A**) breast; (**B**) ovarian; (**C**) lung; (**D**) gastric; and (**D**) liver cancer.

Analysis results from the second server showed that the expression of RACGAP1 negatively influenced OS, distant metastasis-free survival (DMFS), and relapse-free survival (RFS) (*p* < 0.001) (Figure 4A) in breast cancer patients. Regarding ovarian cancer, RACGAP1 was predicted to correlate with poor OS (*p* < 0.01) and progression-free survival (PFS) (*p* < 0.05), but not post-progression survival (PPS) (Figure 4B). Moreover, our analyzed gene was related to poor OS, first progression (F.P.) (*p* < 0.001), and PPS (*p* < 0.01) in lung cancer (Figure 4C). Gastric cancer was also affected by RACGAP1 expression in terms of patients’ survival in relation to OS, PPS (*p* < 0.001), and F.P. (*p* < 0.01) (Figure 4D). Finally, liver cancer demonstrated poor OS, disease-specific survival (DSS), PFS, and RFS (*p* < 0.001) (Figure 4E) with RACGAP1 expression. The results of both databases indicate that an overall poor clinical outcome is expected with RACGAP1 expression in cancer patients.

### 2.4. RACGAP1 Mutation Analysis

**Figure 5:**
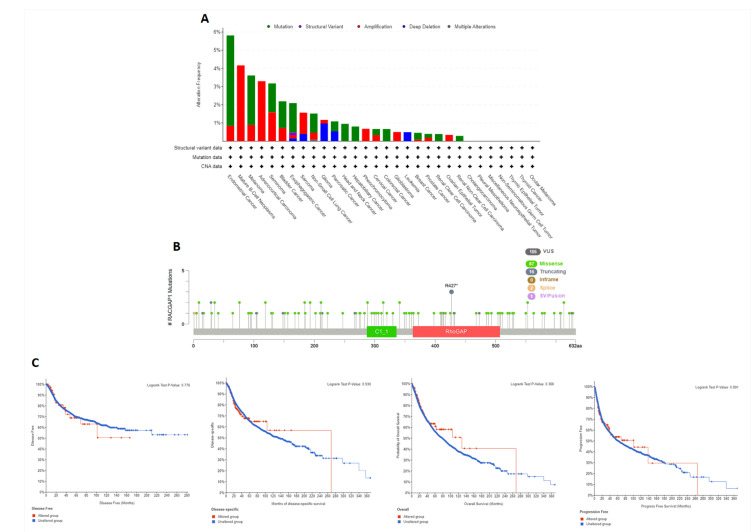
Mutation assessment for RACGAP1 using the cBioPortal tool. (**A**) The alteration frequency with mutation type in a panel of analyzed human cancers; (**B**) a map representation of the sites and types of RACGAP1 mutations; (**C**) assessment of the correlation between RACGAP1 mutation and disease-free, disease-specific, progression-free, and OS.

In the present study, we investigated the copy number alteration (CNA) of RACGAP1 in clinical specimens. Based on the results of the cBioPortal web server, the endometrial cancer subtype had the highest frequency of RACGAP1 mutations, approximately 6%, where the mutation was the predominant form of genetic alterations. With the exception of glioma and leukemia, which exhibited deep deletion as the predominant form of genetic alterations, and pancreatic cancer, which revealed two different forms of alterations (mutation as well as deep deletion) with a comparable percentage of occurrence, it is notable that the majority of the investigated cancers exhibited either “amplification” or “mutation” as the predominant form of genetic alteration (Figure 5A). Subsequently, we investigated the sites and types of RACGAP1 mutations (Figure 5B), where we found 106 total mutations with missense mutations in the first place with 87 recorded samples. Regarding gene location, site R427 was reported to be the most altered site with three mutations (sample with breast invasive ductal carcinoma, second with COAD, and last with LUSC). Finally, we analyzed the correlation between RACGAP1 alteration and patients’ survival, where genetic alteration of RACGAP1 showed no significant difference between altered and unaltered groups in terms of DFS, PFS, OS, and DSS (Figure 5C).

### 2.5. RACGAP1 Phosphorylation Analysis

**Figure 6:**
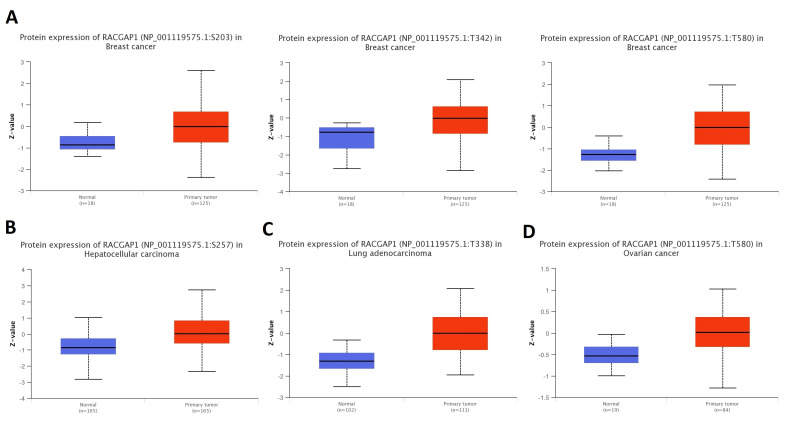
Differential phosphorylation analysis of RACGAP1 in tumor samples versus normal ones. (**A**) Breast cancer; (**B**) hepatocellular carcinoma; (**C**) LUAD; (**D**) ovarian cancer.

We analyzed the connection between RACGAP1 phosphorylation levels and tumorigenesis using phosphorylation analysis. Three phosphorylation sites, including S203, T342, and T580, were considerably elevated in breast cancer compared to normal tissues (Figure 6A). Furthermore, the positions S257, T338, and T580 of RACGAP1 were significantly phosphorylated in tissues of hepatocellular carcinoma, LUAD, and ovarian cancer relative to healthy tissue (Figure 6B–D). In contrast to normal tissues, our investigated locations of RACGAP1 exhibited higher phosphorylation in the investigated malignancies.

### 2.6. RACGAP1 Correlates with Immune Infiltration in Several Tumor Types

**Figure 7:**
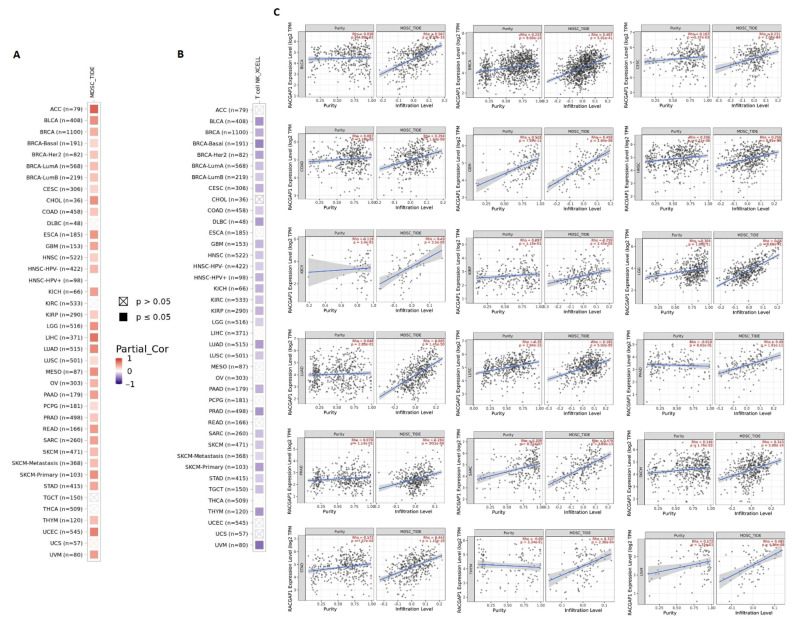
The correlation between RACGAP1 expression level and infiltration of (**A**) myeloid-derived suppressor cells (MDSC) and (**B**) natural killer T cells in a panel of human cancers; (**C**) scatter plots demonstrate the correlation between the expression of RACGAP1 and the infiltration level of MDSC.

First, we tried to find the correlation between RACGAP1 expression and the infiltration of two types of immune cells with opposing roles against tumor growth. Analysis of MDSC infiltration, a cell with immunosuppressive functions in tumor [17], in the panel of TCGA tumors showed that more than 80% of the studied tumors experienced a positive correlation between RACGAP1 expression and MDSC infiltration. Notably, no cancer from the analyzed panel witnessed a negative correlation between RACGAP1 expression and MDSC infiltration (Figure 7A). On the other hand, analysis of NKT cell infiltration, a cell that has a strong antitumor action and was selected as a target for cancer immunotherapy development [18], demonstrated that more than 70% of the analyzed tumors experienced a negative correlation between RACGAP1 expression and NKT cell infiltration (Figure 7B). Again, no cancers in the panel analyzed witnessed a positive correlation between RACGAP1 expression and NKT infiltration. After filtering the results, we found 18 tumors, namely BLCA, BRCA, CESC, COAD, GBM, HNSC, KICH, KIRP, LGG, LUAD, LUSC, PAAD, PRAD, SARC, SKCM, STAD, THYM, and UVM, experiencing a positive correlation between the expression of RACGAP1 and MDSC in addition to a negative correlation between the same gene expression and NKT cell infiltration. The scatter plots that demonstrate the correlation between the expression of RACGAP1 and the infiltration level of MDSC in these 18 filtered tumors are shown in (Figure 7C).

**Figure 8:**
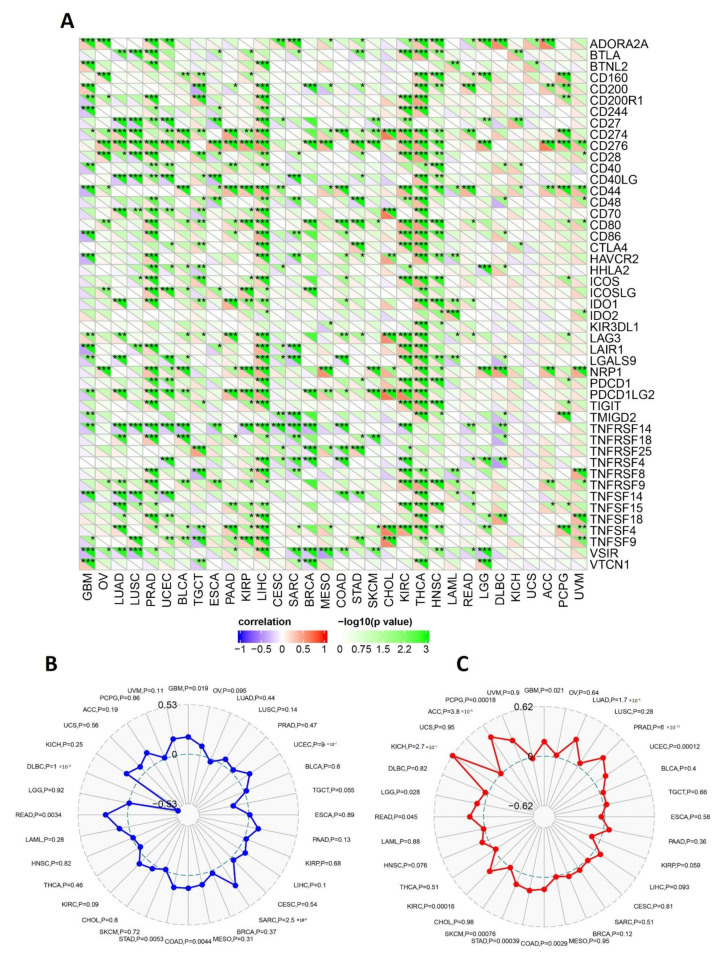
Correlations of RACGAP1 expression with immune checkpoints, MSI, and TMB. (**A**) Heatmap correlating the immune checkpoints and RACGAP1 across a list of human tumors; (**B**) and (**C**) are radar charts showing the overlaps of RACGAP1 with MSI and TMB, respectively; * means statistically significant *p* > 0.05, ** means high statistically significant *p* > 0.001 and *** means *p* > 0.0001.

Following that, the SangerBox online server was employed to find the correlation of RACGAP1 expression with the immune checkpoint, MSI, and TMB. Regarding the immune checkpoint, expressions of RACGAP1 in THCA, KIRC, LIHC, and PRAD were positively correlated with several immune checkpoint genes, while tumors UCS and KICH experienced no significant correlation between our target gene expression and most of the immune checkpoint genes (Figure 8A). In GBM, UCEC, COAD, STAD, and SARC, the expression of RACGAP1 was discovered to be significantly positively associated with MSI. Furthermore, only one tumor, DLBC, revealed a significant negative association between RACGAP1 expression and MSI (Figure 8B). Finally, our target gene expression analysis and TMB showed a significantly positive correlation in ACC, PCPG, GBM, LUAD, PRAD, UCEC, COAD, STAD, SKCM, KIRC, READ, KICH, and LGG (Figure 8C).

### 2.7. Analysis of Interacting and Correlated Proteins to RACGAP1

**Figure 9:**
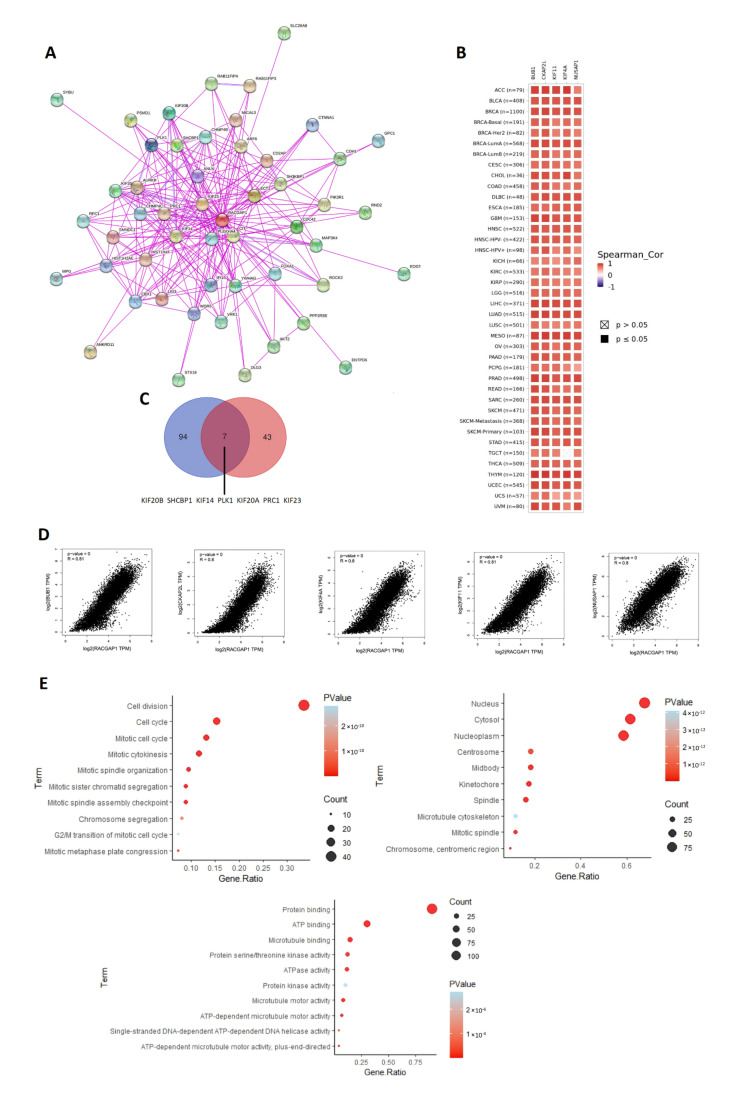
RACGAP1–protein network interactions. (**A**) A map of the top 50 RACGAP1-interacting proteins as determined by the STRING database; (**B**) heatmap for top 5 RACGAP1-correlated proteins in the tumor tissue; (**C**) Venn diagram showing the intersection between RACGAP1-interacting and -correlating proteins; (**D**) expression correlation between RACGAP1 and genes (BUB1, CKAP2L, KIF4A, KIF11, and NUSAP1) as determined by GEPIA2; (**E**) G.O. enrichment analysis based on RACGAP1-binding and interacted genes.

According to the aforementioned findings, RACGAP1 has a significant correlation with the survival of patients with cancer and influences the immune cells in the tumor microenvironment. Therefore, it is essential to investigate the probable molecular pathways of this gene across several cancers. The top 50 experimentally confirmed RACGAP1-interacting proteins were extracted from the STRING database and displayed as a protein–protein interaction network (Figure 9A). Furthermore, the GEPIA2 webserver was explored to obtain the 100 genes correlated to RACGAP1 in the panel of TCGA tumors. The “Correlation Analysis” module was employed to obtain the plots of the top five correlating genes. They were ordered as follows: KIF11 (R = 0.81), BUB1 (R = 0.81), CKAP2L (R = 0.80), KIF4A (R = 0.80), and NUSAP1 (R = 0.80) (Figure 9D). Furthermore, a heatmap created by the “Gene Corr” module at TIMER validated the significant positive correlation between these five genes and RACGAP1 in the complete list of TCGA tumors (with the exception of TGCT, in which the connection with KIF4A was nonsignificant) (Figure 9B). Seven genes, including KIF20B, SHCBP1, KIF14, PLK1, KIF20A, and PRC1, as well as KIF23, were discovered to be duplicated upon the comparison of the two lists created previously (Figure 9C).

After the elimination of duplicates, a unique dataset was created by combining the two lists and uploaded to DAVID for Reactome and Gene Ontology (G.O.) enrichment analyses. Our gene list may be associated with DNA repair, chromatin remodeling, cellular response to DNA damage, and DNA replication, as indicated by the results of the biological process study. In terms of cellular components, the majority of genes were localized in the nucleus and nucleoplasm. Furthermore, the supplied gene list was enriched for protein, RNA, and DNA binding whenever its molecular function was evaluated (Figure 9E). Ultimately, the Reactome pathway analysis revealed that RACGAP1 is strongly associated with the cell cycle and mitotic division (Figure S2).

## 3. Discussion

RACGAP1 is an essential cellular protein that was found to be involved in many cellular processes, including cytokinesis, transformation, invasive migration, and metastasis [19]. Several studies have analyzed the role of RACGAP1 in different types of human cancers. For GIT-related cancers, there was a significant correlation of RACGAP1 protein expression at the invasive front of gastric cancer with older age, tumor size, lymph node metastasis, lymphatic invasion, vascular invasion, and advanced stage [20]. Moreover, higher RACGAP1 expression and Ki-67 index were correlated with unfavorable clinicopathological features in predicting poor outcomes of gastrointestinal stromal tumors [21]. Furthermore, RACGAP1 expression was found to increase malignant tumor potential and was used as a predictive biomarker for lymph node metastasis and poor prognosis in colorectal cancer [22]. Regarding its role in breast cancer progression, RACGAP1 was able to modulate ECT2-dependent mitochondrial quality control to drive breast cancer metastasis [23]. A study on 81 esophageal carcinomas (E.C.) patients showed that RACGAP1 could play a pivotal role in E.C. development, suggesting that it could be used as an indicator of prognosis in E.C. patients [24]. Moving to other types of human tumors, RACGAP1 was able to promote melanoma transendothelial migration through mediating adherens junction disassembly [25] and promoted proliferation and cell cycle progression by regulating CDC25C in cervical cancer cells [26]. RACGAP1 was also found to be a downstream effector of E2F7-dependent resistance to doxorubicin and a prognostic for OS in squamous cell carcinoma [27]. Considering hepatocellular carcinoma, RACGAP1 upregulation was significantly associated with the early recurrence of human hepatocellular carcinoma [28], as it activates the RACGAP1/Rho/ERK signaling axis as a competing endogenous RNA to promote early recurrence of hepatocellular carcinoma [29].

Although several studies have tried to analyze the oncogenic of RACGAP1 in several human cancers, there is a lack of a comprehensive study that can deal with the effect of RACGAP1 from many perspectives in a list of several human tumors. It is already established that the tumor microenvironment is complex, with several factors involved in tumor development, the immune response against this abnormal growth, patients’ response to tumor therapy, and OS [30]. This complex status of the tumor confirms the requirement of a deep approach that can correlate a targeted gene with tumor progression through different analysis points. For this purpose, the current study applied a pan-cancer analysis for the oncogenic behavior of RACGAP1. An important characteristic of the oncogenic proteins is their upregulation in tumor tissue compared to the normal one. It is worth noting that the usage of bioinformatics tools and the exploration of tumor-related databases to study the roles and behavior of a specific gene in a list of human tumors or a targeted specific tumor have been applied in several previous studies. For example, IQGAP3 was investigated and expected to serve as an effective prognostic biomarker for pan-cancer immune-related therapy [31]. Additionally, multiomics studies have revealed CCNB1 and butyrophilins as potential prognostic biomarkers for ACC and breast cancer, respectively [32,33].

For this reason, our study started with the differential expression of RACGAP1 in a list of human tumors where it was found to be significantly upregulated in BLCA, BRCA, CHOL, COAD, ESCA, GBM, HNSC, KICH, KIRC, LIHC, LUAD, LUSC, PRAD, READ, STAD, UCEC, CESC, KIRP, ACC, DLBC, LGG, O.V., SARC, SKCM, THYM, UCS, and PCPG. Following that, our study tried to reveal if there is a relation between RACGAP1 expression and the cancer stage, where we found that ACC, BRCA, KICH, KIRC, KIRP, LIHC, LUAD, LUSC, and UCEC experienced a progression in the tumor stage with RACGAP1 expression. Not only the tumor stage but also tumor metastasis showed a positive correlation with RACGAP1 expression in breast, kidney, and liver cancers. Our last differential comparison was based on the RACGAP1 protein level analysis in normal and tumor tissues. Again, the trend of elevated RACGAP1 expression in tumor tissues was observed in colon cancer, HNCS, LUAD, PAAD, UCEC, ovarian cancer, and hepatocellular carcinoma, where IHC staining, which was high for RACGAP1 in analyzed tumor tissues, confirmed our findings.

Survival analysis is a basic point of investigation for assessing disease progression and the patient’s response to medical treatment [34]. Consequently, the current study aimed to find the correlation between RACGAP1 expression and patients’ survival. The results from the GEPIA database demonstrated a positive correlation between RACGAP1 expression and the poor prognosis in ACC, KIRP, LGG, LIHC, PAAD, SARC, and SKCM in terms of DFS and OS. Furthermore, the output of the KM plot analysis confirmed this positive correlation in all models studied of breast, lung, gastric, and liver cancers, which recommends the use of RACGAP1 as a prognostic biomarker in the above-mentioned tumors. Mutations in several genes were found to be good prognostic markers for human cancer; examples include mutated *KRAS* that was correlated with a poor prognosis of pancreatic [35] and lung cancer [36], and mutated *NRAS* that was associated with a poor prognosis of metastatic melanoma [37]. Therefore, our next survival analysis step was to study whether the RACGAP1 genetic alteration could also affect patients’ survival; we found that RACGAP1 genetic alteration did not significantly correlate with the patient’s survival in four analyzed models of altered and unaltered groups.

The status of gene methylation has been extensively studied in several human cancers. Previous studies generally found that DNA hypermethylation was a major mechanism for silencing tumor suppressor genes [38]. On the other hand, the oncogenes experienced a hypomethylation state as a mechanism for their activation to induce tumor progression [39]; for example, the hypomethylation state for the oncogenes *AQP1*, *LINE-1,* and *ELMO3* was reported in salivary gland adenoid cystic carcinoma [40], colorectal cancer [41], and lung cancer [42], respectively. From this point, we performed a methylation analysis for RACGAP1. As expected, several tumors, including UCEC, COAD, PRAD, BLCA, LIHC, HNSC, TGCT, BRCA, and THCA, showed hypomethylation in tumor samples versus the normal one. Additionally, CpG aggregated methylation data revealed that all of the significant results favored RACGAP1 hypomethylation in the tumor sample versus the normal one (except for CHOL).

Tumor immunotherapy, which witnessed a great evolution in the last few decades, became a well-established approach for fighting against cancer [43] where immune checkpoint inhibitors such as αPD-1 have been approved for the treatment of many types of human cancer, such as malignant melanoma, gastric carcinoma, and hepatocellular carcinoma [44]. For this purpose, it was important to study the correlation between elevated RACGAP1 expression in tumor tissue and the tumor infiltration of different types of immune cells. The first cell analyzed was MDSC, which was found to positively affect tumor cell survival and metastasis [45]. Additionally, it inhibits other cells with fighting ability against growing tumors (CD8 T cells and N.K. cells), supports tumor angiogenesis, and is involved in forming cancer stem cells [46]. Consequently, it was not surprising that the elevated level of MDSC infiltration was correlated with poor clinical outcomes for cancer patients [47]. The current study revealed that ACC, BLCA, BRCA, CESC, COAD, HNSC, HNSC-HPV-, HNSC-HPV+, KIRP, LIHC, LUAD, LUSC, O.V., READ, SARC, SKCM, STAD, TGCT, UCEC, and UCS experienced a positive correlation between RACGAP1 expression and MDSC infiltration. Notably, cytokines such as CCL2, CCL5, and CSF1 were found to be involved in the attraction of MDSCs to the tumor site [48]. However, our finding of a positive association between RACGAP1 expression and MDSC infiltration has not yet been fully investigated. The correlation between RACGAP1 upregulation and specific chemokine expression could be a possible mechanism that might explain this correlation. The second cell that was investigated for its correlation with RACGAP1 upregulation is the NKT cell. This kind of cell demonstrated important roles in fighting against early tumors, where it participates in cancer immune surveillance and secretes several effector molecules [49]. Due to its tumor suppressive roles, NKT cell abundance in the tumor tissue was a positive prognostic factor for patients’ survival in several human cancers [50]. Our results revealed a significant negative correlation between RACGAP1 expression and NKT infiltration in BLCA, BRCA, CESC, COAD, HNSC, HNSC-HPV-, KIRC, KIRP, LUAD, MEOV, PRAD, SKCM, STAD, THYM, and UVM. Another interesting finding is that not even one tumor in our analyzed list experienced a positive between RACGAP1 expression and NKT infiltration. Putting the results of RACGAP1 expression and MDSC and NKT infiltration together, we can conclude that the upregulation of RACGAP1 expression could be used as a marker for a poor immune response against a growing tumor.

MSI and TMB are considered promising biomarkers for the patient’s response to immunotherapy [51], where a robust antitumor effect of αPD1 treatment was observed with colorectal cancer patients with high microsatellite instability (MSI-H) [52]. Similarly, high TMB was positively correlated with a better clinical outcome across diverse tumors [53]. For this purpose, we tried to find a correlation between the upregulation of RACGAP1 in tumor tissue and those promising biomarkers. Our analysis revealed a positive correlation between MSI in READ, LUSC, UCEC, and BRCA and RACGAP1 expression. Additionally, ACC, PRAD, TGCT, LIHC, and READ experienced a positive correlation between RACGAP1 expression and TMB level. Collectively, our findings exposed a research question about the probability of relying on RACGAP1 expression in the above-mentioned tumor as a potential biomarker for patients’ response to tumor immunotherapy.

As a final point of analysis, the current study aimed to investigate the molecular mechanism of RACGAP1 in tumor progression where the top 50 interacting proteins and top 100 correlated proteins to RACGAP1 in the tumor tissue were obtained from the STRING and GEPIA2 databases, respectively, and we interestingly found that PARP1 was a common protein in both of the generated datasets. This protein was a regulator for prostate cancer growth and progression through transcriptional regulatory functions [54]; moreover, it was highly expressed in SCLC, where its knockdown led to SCLC growth inhibition [55]. Moreover, PARP1 was a prognostic biomarker for poor clinical outcomes in breast cancer patients [56]; therefore, PARP1 inhibitors were extensively studied as a promising class of anticancer agents [57]. Analysis of RACGAP1 correlated proteins in the tumor tissue revealed that POLR3C, PRKAB2, SETDB1, GPATCH4, and MSTO1 were the top five. SETDB1 was implicated as an oncogene in several human tumors [58], where it was involved in tumor progression in HCC through the methylation of p53 [59]. Furthermore, SETDB1 has been involved in NSCLC progression through WNT–β-catenin pathway stimulation, and for these roles, it was nominated to be a therapeutic target to fight against numerous cancers [60]. It is noteworthy that the complex oncogenic functions of POLR3C, PRKAB2, SETDB1, and GPATCH4 have not been studied yet, and because of being from the top correlating proteins with RACGAP1 in tumor tissues, their potential oncogenic roles and interacting network should be further studied to present clues for novel tumor treatment strategies.

## 4. Materials and Methods

### 4.1. Gene Expression Analysis

Initially, data collected from the Tumor Immune Estimation Resource, version 2 (TIMER2.0) were used to visualize the level of RACGAP1 expression in various cancers compared to normal tissue [56], where the list of tumors of the Cancer Genome Atlas (TCGA) (Supplementary Table S1) was employed for this comparison. Following TIMER2.0 analysis, it was discovered that several cancers lacked normal tissue for comparisons. Subsequently, RACGAP1 expression in healthy and malignant tissue was compared in several human cancers using the Gene Expression Profiling Interactive Analysis (GEPIA) database (http://gepia.cancer-pku.cn/index.html (accessed on 20 September 2022)) [57]. We used the TISBID website to examine the connection between the expression of the RACGAP1 gene and the grading of the tumor [58]. Last but not least, the expression of RACGAP1 in the tumor, normal, and metastatic tissues was evaluated using the TNMplot (differential gene expression analysis in Tumor, Normal, and Metastatic tissues) online server and its Kruskal–Wallis test for significance testing [59].

### 4.2. Protein Expression and Immunohistochemistry (IHC) Staining

To examine the expression patterns of RACGAP1 protein between normal and malignant specimens, the UALCAN program, which conducts protein expression analysis using data collected from the Clinical Proteomic Tumor Analysis Consortium (CPTAC), was utilized [60]. In addition, we utilized HPA to track IHC images of RACGAP1 expression in normal and malignant tissues for tumors, in which UALCAN analysis revealed significant differences in order to validate our findings.

### 4.3. Survival Prognosis Analysis

The GEPIA2.0 webserver was initially utilized to evaluate survival outcomes. We tested RACGAP1 in the “survival analysis” section, chose the whole tumor list from the TCGA cohort, and acquired the heatmap for the two possible approaches from the server (OS and disease-free survival). Then, we analyzed the relationship between RACGAP1 expression as well as survival rates in five different cancers using the KM plotter [61]. (Breast, ovarian, lung, gastric, and liver cancer).

### 4.4. Gene Alteration Analysis

We utilized the cBioPortal web server to conduct an extensive and comprehensive investigation of RACGAP1 polymorphisms [62]. In order to perform this study, we chose “TCGA PanCancer Atlas Studies” as our data source and followed three key guidelines. The “cancer types summary” tab was used to retrieve the mutation frequency as well as the mutation type results. Furthermore, the “Mutations” tab was utilized to view RACGAP1 alteration sites. We visited the “Comparison/Survival” page to examine the relationship between RACGAP1 genetic alterations and survival rates.

### 4.5. RACGAP1 Phosphorylation Analysis

Numerous enzymes and receptors are activated/deactivated by phosphorylation and dephosphorylation processes, which makes protein phosphorylation a significant physiological regulation mechanism [63]. Using data retrieved from the Clinical Proteomic Tumor Study Consortium, we performed a protein phosphorylation investigation of RACGAP1 in malignant tissues versus normal tissues using the UALCAN software (CPTAC).

### 4.6. Immune Reactivity Assessment

Initially, we utilized the TIMER2 web server [64] to determine the association between RACGAP1 expression patterns and immune cells that could also play positive or negative roles in the development of TCGA cancers. We inserted the name of our target gene into the “gene” module within the “immune” partition. We chose two invading immune cell types, including myeloid-derived suppressor cells (MDSCs) as well as natural killer T cells (NKT), having opposing functions in tumor formation. Subsequently, we generated heatmaps and scatter plots to illustrate our investigated correlations. Then, we accessed the SangerBox web server to investigate the relationship between our target gene and three variables: immunological checkpoints, microsatellite instability (MSI), and tumor mutational burden (TMB).

### 4.7. Protein–Protein Interaction and Enrichment Analysis

A total of two web servers were assessed in order to investigate the proteins that may have significant interactions or correlations with RACGAP1. To generate the network of RACGAP1-interacting proteins, we subsequently consulted the STRING database [65]. Setting “Experiments” as the active interaction source and “low confidence” as the interaction score yielded the top 50 interacting proteins. The “Correlation Analysis” module on the same database and the “Gene Corr” module in TIMER were employed to generate the correlation curves and the heatmap for the top five associated genes. Then, we accessed the online server (http://bioinformatics.psb.ugent.be/webtools/Venn/ (accessed on 20 September 2022)) in order to identify the proteins shared by both the “RACGAP1 interacting” and “RACGAP1 correlating” lists. After integrating both lists and deleting duplicate records, the resulting set of data was uploaded to the Database of Annotation, Visualization, and Integrated Discovery (DAVID) [66] for functional enrichment analysis, with the findings shown utilizing “ggplot2” package of R. (4.2.0).

## 5. Conclusions

RACGAP1 is an oncogene that was shown to be overexpressed as mRNA and protein in a significant number of human malignancies, and its overexpression was discovered to be correlated with poor health outcomes. RACGAP1 expression was positively correlated with immunosuppressive cell (MDSC) infiltration. On the contrary, the gene was adversely associated with the invasion of tumor-fighting cells (NKT cells). Furthermore, TMB and MSI were connected with RACGAP1 expression in various human malignancies; hence, these results could designate RACGAP1 as a reliable prognostic biomarker, a marker for patients’ responsiveness to immunotherapy, and a promising target for cancer therapeutics. Moreover, the current study explored the interaction network of RACGAP1 with other proteins in the tumor microenvironment, where future deep investigation of our primary data can explain the mechanism of RACGAP1 in the induction of tumor progression and open the avenue for the development of novel antitumor treatment.

## Data Availability

Data available upon being requested.

## Supplementary Materials

The following supporting information can be downloaded at: https://www.mdpi.com/article/10.3390/ijms232214102/s1.

## References

[1] Gharib A.F., Eldeen M.A., Khalifa A.S., Elsawy W.H., Eed E.M., El Askary A., Eid R.A., Soltan M.A., Raafat N. (2021). Assessment of Glutathione Peroxidase-1 (GPX1) Gene Expression as a Specific Diagnostic and Prognostic Biomarker in Malignant Pleural Mesothelioma. Diagnostics.

[2] Mahaboob Batcha A.T., Subramaniam G., Venkatachalam K. (2022). Purified Banana lectin (BanLec) isolated from the ripen pulp of Musa Paradisiaca induces apoptosis in cancer cell lines: In vitro study. Adv. Tradit. Med..

[3] Hnisz D., Weintrau A.S., Day D.S., Valton A.-L., Bak R.O., Li C.H., Goldmann J., Lajoie B.R., Fan Z.P., Sigova A.A. (2016). Activation of proto-oncogenes by disruption of chromosome neighborhoods. Science.

[4] Blum A., Wang P., Zenklusen J.C. (2018). SnapShot: TCGA-Analyzed Tumors. Cell.

[5] Tomczak K., Czerwińska P., Wiznerowicz M. (2015). The Cancer Genome Atlas (TCGA): An immeasurable source of knowledge. Wspolczesna Onkol..

[6] Lee J.-S., Kamijo K., Ohara N., Kitamura T., Miki T. (2003). MgcRacGAP regulates cortical activity through RhoA during cytokinesis. Exp. Cell Res..

[7] Zhao W.-M., Fang G. (2005). MgcRacGAP controls the assembly of the contractile ring and the initiation of cytokinesis. Proc. Natl. Acad. Sci. USA.

[8] Jantsch-Plunger V., Gönczy P., Romano A., Schnabel H., Hamill D., Schnabel R., Hyman A., Glotzer M. (2000). CYK-4: A Rho family GTPase activating protein (GAP) required for central spindle formation and cytokinesis. J. Cell Biol..

[9] Kawashima T., Bao Y.C., Minoshima Y., Nomura Y., Hatori T., Hori T., Fukagawa T., Fukada T., Takahashi N., Nosaka T. (2009). A Rac GTPase-Activating Protein, MgcRacGAP, Is a Nuclear Localizing Signal-Containing Nuclear Chaperone in the Activation of STAT Transcription Factors. Mol. Cell. Biol..

[10] Sahai E. (2005). Mechanisms of cancer cell invasion. Curr. Opin. Genet. Dev..

[11] Mishima M., Kaitna S., Glotzer M. (2002). Central Spindle Assembly and Cytokinesis Require a Kinesin-like Protein/RhoGAP Complex with Microtubule Bundling Activity. Dev. Cell.

[12] Touré A., Morin L., Pineau C., Becq F., Dorseuil O., Gacon G. (2001). Tat1, a Novel Sulfate Transporter Specifically Expressed in Human Male Germ Cells and Potentially Linked to RhoGTPase Signaling. J. Biol. Chem..

[13] Naud N., Toure A., Liu J., Pineau C., Morin L., Dorseuil O., Escalier D., Chardin P., Gacon G. (2003). Rho family GTPase Rnd2 interacts and co-localizes with MgcRacGAP in male germ cells. Biochem. J..

[14] Zhang P., Bai H., Fu C., Chen F., Zeng P., Wu C., Ye Q., Dong C., Song Y., Song E. (2015). RacGAP1-driven focal adhesion formation promotes melanoma transendothelial migration through mediating adherens junction disassembly. Biochem. Biophys. Res. Commun..

[15] Strimpakos A., Sampaziotis D., Psyrri A. (2018). RACGAP1 (Rac GTPase activating protein 1). Atlas Genet. Cytogenet. Oncol. Haematol..

[16] Gabrilovich D.I., Nagaraj S. (2009). Myeloid-derived suppressor cells as regulators of the immune system. Nat. Rev. Immunol..

[17] Liu X., Li L., Si F., Huang L., Zhao Y., Zhang C., Hoft D.F., Peng G. (2021). NK and NKT cells have distinct properties and functions in cancer. Oncogene.

[18] Yamazaki D., Kurisu S., Takenawa T. (2009). Involvement of Rac and Rho signaling in cancer cell motility in 3D substrates. Oncogene.

[19] Saigusa S., Tanaka K., Mohri Y., Ohi M., Shimura T., Kitajima T., Kondo S., Okugawa Y., Toiyama Y., Inoue Y. (2015). Clinical significance of RacGAP1 expression at the invasive front of gastric cancer. Gastric Cancer.

[20] Sahin S., Ekinci O., Seckin S., Dursun A. (2017). Proliferation markers RacGAP1 and Ki-67 in gastrointestinal stromal tumors by immunohistochemistry with respect to clinicopathological features and different risk stratification systems. Int. J. Clin. Exp. Pathol..

[21] Imaoka H., Toiyama Y., Saigusa S., Kawamura M., Kawamoto A., Okugawa Y., Hiro J., Tanaka K., Inoue Y., Mohri Y. (2014). RacGAP1 expression, increasing tumor malignant potential, as a predictive biomarker for lymph node metastasis and poor prognosis in colorectal cancer. Carcinogenesis.

[22] Ren K., Zhou D., Wang M., Li E., Hou C., Su Y., Zou Q., Zhou P., Liu X. (2021). RACGAP1 modulates ECT2-Dependent mitochondrial quality control to drive breast cancer metastasis. Exp. Cell Res..

[23] Yin C., Toiyama Y., Okugawa Y., Shigemori T., Yamamoto A., Ide S., Kitajima T., Fujikawa H., Yasuda H., Okita Y. (2019). Rac GTPase-Activating Protein 1 (RACGAP1) as an Oncogenic Enhancer in Esophageal Carcinoma. Oncology.

[24] Ruan X., Jiang J. (2022). RACGAP1 promotes proliferation and cell cycle progression by regulating CDC25C in cervical cancer cells. Tissue Cell.

[25] Hazar-Rethinam M., de Long L.M., Gannon O.M., Boros S., Vargas A.C., Dzienis M., Mukhopadhyay P., Saenz-Ponce N., Dantzic D.D., Simpson F. (2015). RacGAP1 Is a Novel Downstream Effector of E2F7-Dependent Resistance to Doxorubicin and Is Prognostic for Overall Survival in Squamous Cell Carcinoma. Mol. Cancer Ther..

[26] Wang S.M., Ooi L.L.P., Hui K.M. (2011). Upregulation of Rac GTPase-Activating Protein 1 Is Significantly Associated with the Early Recurrence of Human Hepatocellular Carcinoma. Clin. Cancer Res..

[27] Wang M.-Y., Chen D.-P., Qi B., Li M.-Y., Zhu Y.-Y., Yin W.-J., He L., Yu Y., Li Z.-Y., Lin L. (2019). Pseudogene RACGAP1P activates RACGAP1/Rho/ERK signalling axis as a competing endogenous RNA to promote hepatocellular carcinoma early recurrence. Cell Death Dis..

[28] Zabady S., Mahran N., Soltan M.A., Eldeen M.A., Eid R.A., Albogami S., Fayad E., Matboli M., Habib E.K., Hasanin A.H. (2022). Cyanidin-3-Glucoside Modulates hsa_circ_0001345/miRNA106b/ATG16L1 Axis Expression as a Potential Protective Mechanism against Hepatocellular Carcinoma. Curr. Issues Mol. Biol..

[29] Nagy Á., Munkácsy G., Győrffy B. (2021). Pancancer survival analysis of cancer hallmark genes. Sci. Rep..

[30] Buscail L., Bournet B., Cordelier P. (2020). Role of oncogenic KRAS in the diagnosis, prognosis and treatment of pancreatic cancer. Nat. Rev. Gastroenterol. Hepatol..

[31] Shen H., Che K., Cong L., Dong W., Zhang T., Liu Q., Du J. (2017). Diagnostic and prognostic value of blood samples for KRAS mutation identification in lung cancer: A meta-analysis. Oncotarget.

[32] Jakob J.A., Bassett R.L., Ng C.S., Curry J.L., Joseph R.W., Rohlfs M.L., Alvarado G.C., Richard J., Gershenwald J.E., Kim K.B. (2012). NRAS mutation status is an independent prognostic factor in metastatic melanoma. Cancer.

[33] Anglim P.P., A Alonzo T., A Laird-Offringa I. (2008). DNA methylation-based biomarkers for early detection of non-small cell lung cancer: An update. Mol. Cancer.

[34] Romero-Garcia S., Prado-Garcia H., Carlos-Reyes A. (2020). Role of DNA Methylation in the Resistance to Therapy in Solid Tumors. Front. Oncol..

[35] Shao C., Sun W., Tan M., Glazer C.A., Bhan S., Zhong X., Fakhry C., Sharma R., Westra W.H., Hoque M.O. (2011). Integrated, Genome-Wide Screening for Hypomethylated Oncogenes in Salivary Gland Adenoid Cystic Carcinoma. Clin. Cancer Res..

[36] Hur K., Cejas P., Feliu J., Moreno-Rubio J., Burgos E., Boland C.R., Goel A. (2013). Hypomethylation of long interspersed nuclear element-1 (LINE-1) leads to activation of proto-oncogenes in human colorectal cancer metastasis. Gut.

[37] Søes S., Daugaard I.L., Sørensen B.S., Carus A., Mattheisen M., Alsner J., Overgaard J., Hager H., Hansen L.L., Kristensen L.S. (2014). Hypomethylation and increased expression of the putative oncogene ELMO3 are associated with lung cancer development and metastases formation. Oncoscience.

[38] Peng M., Mo Y., Wang Y., Wu P., Zhang Y., Xiong F., Guo C., Wu X., Li Y., Li X. (2019). Neoantigen vaccine: An emerging tumor immunotherapy. Mol. Cancer.

[39] Chang E., Pelosof L., Lemery S., Gong Y., Goldberg K.B., Farrell A.T., Keegan P., Veeraraghavan J., Wei G., Blumenthal G.M. (2021). Systematic Review of PD-1/PD-L1 Inhibitors in Oncology: From Personalized Medicine to Public Health. Oncologist.

[40] Condamine T., Ramachandran I., Youn J.-I., Gabrilovich D.I. (2015). Regulation of Tumor Metastasis by Myeloid-Derived Suppressor Cells. Annu. Rev. Med..

[41] Weber R., Fleming V., Hu X., Nagibin V., Groth C., Altevogt P., Utikal J., Umansky V. (2018). Myeloid-Derived Suppressor Cells Hinder the Anti-Cancer Activity of Immune Checkpoint Inhibitors. Front. Immunol..

[42] Zhang S., Ma X., Zhu C., Liu L., Wang G., Yuan X. (2016). The Role of Myeloid-Derived Suppressor Cells in Patients with Solid Tumors: A Meta-Analysis. PLoS ONE.

[43] Kumar V., Patel S., Tcyganov E., Gabrilovich D.I. (2016). The Nature of Myeloid-Derived Suppressor Cells in the Tumor Microenvironment. Trends Immunol..

[44] Bae E.-A., Seo H., Kim I.-K., Jeon I., Kang C.-Y. (2019). Roles of NKT cells in cancer immunotherapy. Arch. Pharm. Res..

[45] Wolf B.J., Choi J.E., Exley M.A. (2018). Novel Approaches to Exploiting Invariant NKT Cells in Cancer Immunotherapy. Front. Immunol..

[46] Zhao Z., Li W., Zhang X., Ge M., Song C. (2020). Correlation between TMB and MSI in patients with solid tumors. J. Clin. Oncol..

[47] Diaz L.A., Marabelle A., Delord J.-P., Shapira-Frommer R., Geva R., Peled N., Kim T.W., Andre T., Van Cutsem E., Guimbaud R. (2017). Pembrolizumab therapy for microsatellite instability high (MSI-H) colorectal cancer (CRC) and non-CRC. J. Clin. Oncol..

[48] Goodman A.M., Kato S., Bazhenova L., Patel S.P., Frampton G.M., Miller V., Stephens P.J., Daniels G.A., Kurzrock R. (2017). Tumor Mutational Burden as an Independent Predictor of Response to Immunotherapy in Diverse Cancers. Mol. Cancer Ther..

[49] Schiewer M.J., Goodwin J.F., Han S., Brenner J.C., Augello M.A., Dean J.L., Liu F., Planck J.L., Ravindranathan P., Chinnaiyan A.M. (2012). Dual Roles of PARP-1 Promote Cancer Growth and Progression. Cancer Discov..

[50] Byers L.A., Wang J., Nilsson M.B., Fujimoto J., Saintigny P., Yordy J., Giri U., Peyton M., Fan Y.H., Diao L. (2012). Proteomic Profiling Identifies Dysregulated Pathways in Small Cell Lung Cancer and Novel Therapeutic Targets Including PARP. Cancer Discov..

[51] Mazzotta A., Partipilo G., De Summa S., Giotta F., Simone G., Mangia A. (2015). Nuclear PARP1 expression and its prognostic significance in breast cancer patients. Tumor Biol..

[52] Liang H., Tan A.R. (2010). Iniparib, a PARP1 inhibitor for the potential treatment of cancer, including triple-negative breast cancer. IDrugs Investig. Drugs J..

[53] Lazaro-Camp V.J., Salari K., Meng X., Yang S. (2021). SETDB1 in cancer: Overexpression and its therapeutic implications. Am. J. Cancer Res..

[54] Fei Q., Shang K., Zhang J., Chuai S., Kong D., Zhou T., Fu S., Liang Y., Li C., Chen Z. (2015). Histone methyltransferase SETDB1 regulates liver cancer cell growth through methylation of P53. Nat. Commun..

[55] Sun Q.-Y., Ding L.-W., Xiao J.-F., Chien W., Lim S.-L., Hattori N., Goodglick L., Chia D., Mah V., Alavi M. (2014). SETDB1 accelerates tumourigenesis by regulating the WNT signalling pathway. J. Pathol..

[56] Li T., Fu J., Zeng Z., Cohen D., Li J., Chen Q., Li B., Liu X.S. (2020). TIMER2.0 for analysis of tumor-infiltrating immune cells. Nucleic Acids Res..

[57] Tang Z., Li C., Kang B., Gao G., Li C., Zhang Z. (2017). GEPIA: A web server for cancer and normal gene expression profiling and interactive analyses. Nucleic Acids Res..

[58] Ru B., Wong C.N., Tong Y., Zhong J.Y., Zhong S.S.W., Wu W.C., Chu K.C., Wong C.Y., Lau C.Y., Chen I. (2019). TISIDB: An integrated repository portal for tumor–immune system interactions. Bioinformatics.

[59] Bartha Á., Győrffy B. (2021). TNMplot.com: A Web Tool for the Comparison of Gene Expression in Normal, Tumor and Metastatic Tissues. Int. J. Mol. Sci..

[60] Chandrashekar D.S., Bashel B., Balasubramanya S.A.H., Creighton C.J., Ponce-Rodriguez I., Chakravarthi B.V.S.K., Varambally S. (2017). UALCAN: A portal for facilitating tumor subgroup gene expression and survival analyses. Neoplasia.

[61] Lánczky A., Győrffy B. (2021). Web-Based Survival Analysis Tool Tailored for Medical Research (KMplot): Development and Implementation. J. Med. Internet Res..

[62] Gao J., Aksoy B.A., Dogrusoz U., Dresdner G., Gross B.E., Sumer S.O., Sun Y., Jacobsen A., Sinha R., Larsson E. (2013). Integrative Analysis of Complex Cancer Genomics and Clinical Profiles Using the cBioPortal. Sci. Signal..

[63] Ardito F., Giuliani M., Perrone D., Troiano G., Lo Muzio L. (2017). The crucial role of protein phosphorylation in cell signaling and its use as targeted therapy (Review). Int. J. Mol. Med..

[64] Li B., Severson E., Pignon J.-C., Zhao H., Li T., Novak J., Jiang P., Shen H., Aster J.C., Rodig S. (2016). Comprehensive analyses of tumor immunity: Implications for cancer immunotherapy. Genome Biol..

[65] Szklarczyk D., Gable A.L., Nastou K.C., Lyon D., Kirsch R., Pyysalo S., Doncheva N.T., Legeay M., Fang T., Bork P. (2021). The STRING database in 2021: Customizable protein–protein networks, and functional characterization of user-uploaded gene/measurement sets. Nucleic Acids Res..

[66] Sherman B.T., Hao M., Qiu J., Jiao X., Baseler M.W., Lane H.C., Imamichi T., Chang W. (2022). DAVID: A web server for functional enrichment analysis and functional annotation of gene lists (2021 update). Nucleic Acids Res..

