# Supplementary figures and images for: Assessment of RACGAP1 as a Prognostic and Immunological Biomarker in Multiple Human Tumors: A Multiomics Analysis
Source: Int J Mol Sci. 2022 Nov 15;23(22):14102. doi: 10.3390/ijms232214102 (PMC9695706; doi:10.3390/ijms232214102)

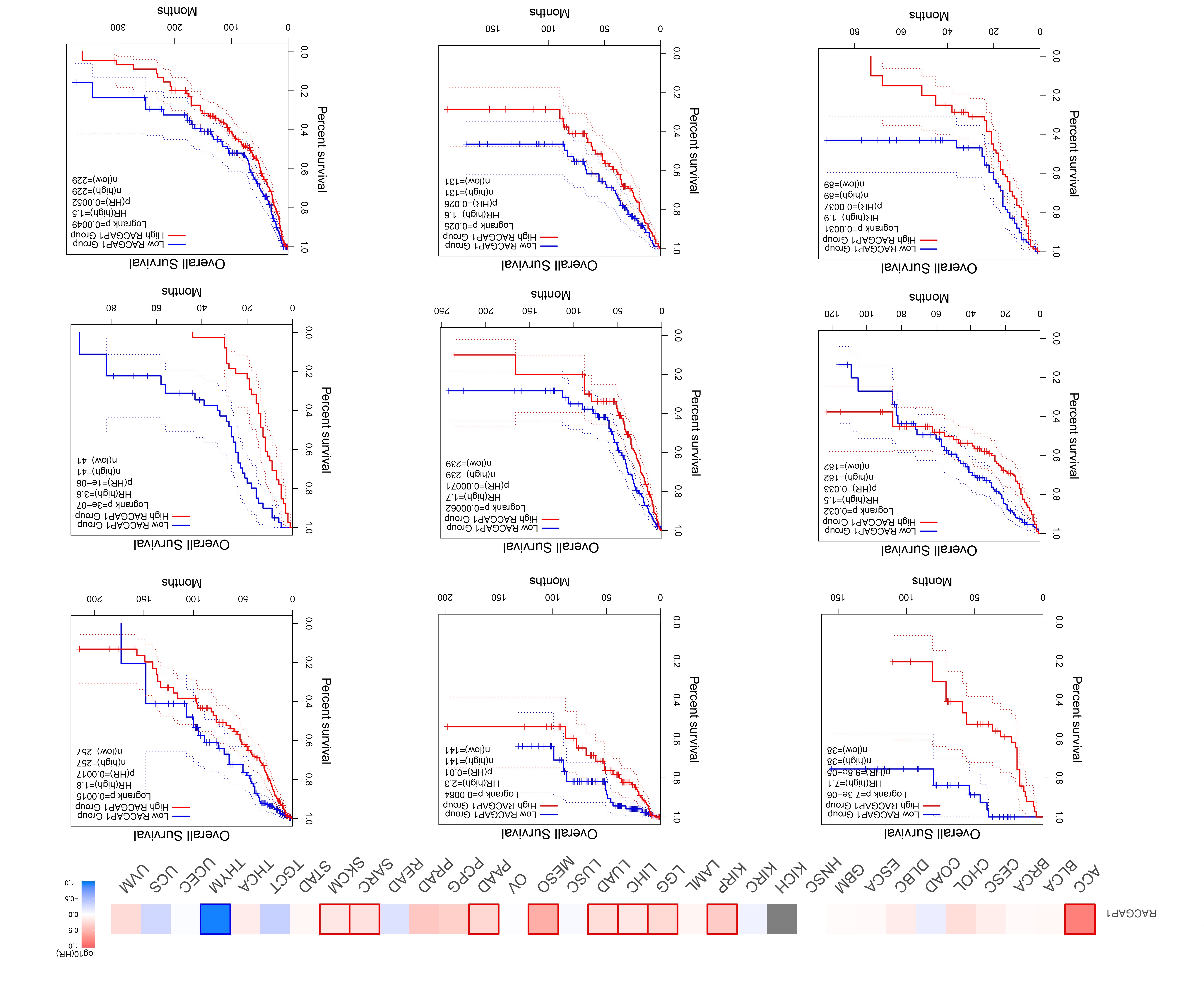

Supplement: Supplementary file 1 [file ijms-23-14102-s001.zip › Supplementary Figure S1.jpg]

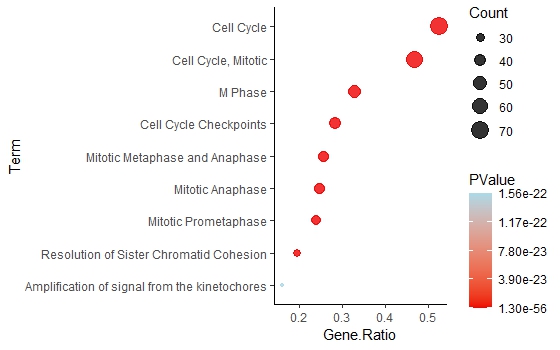

Supplement: Supplementary file 1 [file ijms-23-14102-s001.zip › Supplementary Figure S2.jpeg]
